# Knowledge, Perception, and Practice of Safe Medical Circumcision on HIV Infection Risk Reduction among Undergraduate Students of a Public University in Northern Uganda: A Cross-Sectional Study

**DOI:** 10.1155/2024/1534139

**Published:** 2024-04-09

**Authors:** Lucky Akugizibwe, Deo Benyumiza, Catherine Nekesa, Edward Kumakech, Eustes Kigongo, Nasser Ashaba, Amir Kabunga, Raymond Tumwesigye

**Affiliations:** ^1^Department of Environmental Health and Disease Control, Faculty of Public Health, Lira University, P.O. Box 1035 Lira City, Uganda; ^2^Department of Midwifery, Faculty of Nursing and Midwifery, Lira University, P.O. Box 1035 Lira City, Uganda; ^3^Department of Psychiatry, Faculty of Medicine, Lira University, P.O. Box 1035 Lira City, Uganda; ^4^Department of Emergency Nursing and Critical Care, Faculty of Nursing and Midwifery, Lira University, P.O. Box 1035 Lira City, Uganda

## Abstract

**Background:**

About 70% (25.6 million) of the global HIV/AIDS burden is from Sub-Saharan Africa. Safe male circumcision (SMC) is one of the measures that were adopted by the Ugandan government aimed at reducing the risk of HIV infection contraction, as recommended by the WHO. Its main goal was to maximize HIV prevention impact with voluntary medical circumcision services to all adult men and adolescent boys. The objective of our study was to assess the knowledge, perception, and practice of safe medical circumcision on HIV infection risk reduction among undergraduate students of a public university in Northern Uganda.

**Methods:**

We conducted a cross-sectional survey among 556 randomly selected Lira University undergraduate students from March 2023 to June 2023. With the use of a self-administered questionnaire, we collected data on the knowledge and perceptions of undergraduate students towards safe medical circumcision. Data were exported to Stata® 17 statistical software. Univariate, bivariate, and multivariate regression analyses were done at a statistical level of significance *P* value < 0.05.

**Results:**

Our 556 study participants had an age range of 21-25 years. The majority (81.29%) of the respondents knew that safe medical circumcision reduces the risk of acquiring HIV. However, the perception is that close to 3 in 4 (74.46%) of the students were unsure if they would opt for safe medical circumcision as risk reduction measure against HIV. The practice of safe medical circumcision was 64.8% among the study participants.

**Conclusions:**

More than three in four of the undergraduate students have knowledge on safe medical circumcision as risk reduction measure for HIV infection. And close to 3 in 4 (74.46%) of the student's perception were unsure if they would opt for safe medical circumcision as risk reduction measure against HIV. The practice of safe medical circumcision was 64.8% among the study participants. Therefore, in an effort to increase SMC's adoption for HIV/AIDS prevention, the Ministry of Health of Uganda and related stakeholders in health should work hand in hand with university study bodies in order to optimize SMC uptake among university students.

## 1. Introduction

Globally, 3.4 million young people aged 15-24 years live with HIV/AIDS, and Sub-Saharan Africa carries a significantly high burden [1]. In an attempt to meet the world's commitment to ending the AIDS epidemic by 2030 [2], the WHO recommended SMC as one of the HIV prevention measures. The Ugandan government expected to have circumcised 6.9 million men aged 10-49 by 2020, though by 2016, only 2.5 million men had circumcised and there has never been a great mass safe male circumcision as it was in 2014 [3]. Recent studies have shown that there is a very high increase in the prevalence of HIV among the youth aged 20–24 years in Uganda standing at 10.8% [4], almost doubling the national prevalence of HIV in Uganda at 6.2% [5]. Most undergraduate university students are of this age bracket of 20–24 years and therefore present with a high risk of acquiring HIV infection.

There is a high HIV prevalence in the Northern Uganda region with a 7.2% prevalence of HIV compared to the national prevalence of 6.2% [5]. There is also a growing number of males opting out of circumcision since 2016 up to today [3]; however, younger males remain reluctant to undertake this voluntary initiative. This coupled with the limited numbers of young people undertaking HIV testing and other sexual and reproductive health services in Northern Uganda of about 43% and 42%, respectively, poses a significant challenge in the fight against HIV/AIDS and other sexually transmitted infections [6, 7]. SMC uptake can be attributed to the knowledge about the relevance of safe male circumcision or due to the cultural, social, and religious perceptions within the different societies.

Few studies have so far been conducted on the knowledge and perceptions about SMC among males in high institutions of learning in Uganda, and yet, studies have continued to indicate a higher prevalence of HIV among university students [8, 9]. Given the fact that university students come from all the regions of Uganda, this study is more relevant as it will give a proper understanding of these groups of people on SMC. This study therefore assessed the knowledge, perception, and practice of safe medical circumcision on HIV infection risk reduction among undergraduate students of a public university in Northern Uganda.

## 2. Materials and Methods

### 2.1. Study Design and Setting

The study employed a cross-sectional study design using quantitative methods of data collection and analysis to assess the knowledge, perception, and practice of safe medical circumcision as HIV infection risk reduction among undergraduate students of a public university in Northern Uganda.

This study was at Lira University. Lira University is located in Lira City, Northern Uganda. Lira University is one of the nine public universities in Uganda. Lira City is 339 km by road northwest of Kampala City, the capital of Uganda.

### 2.2. Study Population

This study was conducted among undergraduate students at Lira University. Eligible male undergraduate students enrolled in any academic program of Lira University who were available in the university premises during time for data collection were consented, and those who accepted to take part in this study were issued with a self-administered questionnaire a tool used to collect data from the participants in this study.

### 2.3. Sample Size Estimation

Since the sample space was known, the Yamane formula (1967) was adopted to determine the sample size of the study, that is,

(1) $$\begin{array}{c} n=\frac{N}{1+N\left(e^{2}\right)}, \end{array}$$

where *n* is the sample size, *N* is the size of the population (662), and *e* is an error at a confidence interval of 95% (*e* = 0.05).

Given the total population of undergraduate students at Lira University, which was 1179, with 662 being male, the calculated sample size was 249 from the following calculation:

(2) $$\begin{array}{c} n=\frac{662}{1+662\left(0.05^{2}\right)}, \end{array}$$

where *n* = 249 is the respondents.

Considering a 10% nonresponse rate, the final sample size was determined to be 274 participants. By factoring the design effect (DE) of 2, the estimated sample size was doubled to 548.

### 2.4. Data Collection Procedure

The study participants were selected using consecutive sampling technique. During consecutive sampling, every subject meeting the inclusion criteria is selected until the required sample size is achieved. The data collection process utilized self-administered questionnaires, which were tested through a pilot study to ensure their effectiveness, and the tool had a content validation index of 7.8. The self-administered questionnaire enables the researcher to collect information on patient demographic characteristics and the three dependent variables, that is, knowledge of safe medical circumcision on HIV infection risk reduction, perception of study participants on safe medical circumcision on HIV infection risk reduction, and practice of safe medical circumcision. Eligible male student respondents at Lira University received questionnaires for filling after a clear explanation of the study's purpose and data collection procedures. Each respondent was provided and responded to written informed consent forms before answering the questionnaire.

### 2.5. Data Management and Analysis

Every questionnaire was checked for completeness at the end of each collection day. A data entry screen was created in excel with checks to ensure accuracy during data entry. Data were scanned for out-of-range and missing values before commencing data analysis. The data set was exported and analyzed using Stata version 17. Univariate analysis was done, and descriptive statistics were presented as percentages and frequencies in appropriate tables and pie-chart. At bivariate analysis, associations were established for the individual primary outcomes of this study (knowledge, perception, and practice) using a chi-square test. The level of statistical significance was determined at 95% confidence interval which was *P* < 0.05 at bivariate analysis.

## 3. Results

### 3.1. Sociodemographic Characteristics

In the study, a total of 556 respondents participated, and the majority of participants were aged 21-25 years (80.58%) and academic year two and year three with 33.09%, and the faculties of education and management science had the most significant number of participants, accounting for 29.14% and 26.26% of the total respondents, respectively. Catholics were the dominant religion among the participants, comprising 33.45% of the respondents. Additionally, a large majority of the participants (87.05%) were aware of the health facility where safe male circumcision (SMC) is performed. Details are presented in Table 1.

### 3.2. Knowledge about Safe Male Circumcision

The majority (81.29%) of the respondents knew that circumcision reduces the risk of acquiring HIV, while 56.12% indicated that circumcision of a man does not protect his partner, yet 85.61% accepted that it is easy to get HIV when uncircumcised. The majority (31.29%) disagreed that circumcised men can have unprotected sex and do not get HIV, whereas the bigger portion (55.4%) strongly agreed that all men age groups are eligible for circumcision. On another hand, close to 9 in 10 (88.49%) indicated that a health facility is the best place to conduct SMC. More of the respondents (84.89%) had heard about SMC in the last 12 months, and hygiene was the most common reason (40.29%) for getting circumcised as shown in Table 2.

### 3.3. Association of SMC Knowledge on HIV Risk Reduction and Practice of SMC

All variables at 95% CI were found to be significant consisting of the following: circumcision reduces risk of HIV (*X*^2^ = 18.404, *P* < 0.001), circumcision of a man does not protect his partner from HIV (*X*^2^ = 17.037, *P* < 0.001), it is easy to get HIV when uncircumcised (*X*^2^ = 18.650, *P* < 0.001), circumcised men can have live sex and do not get HIV (*X*^2^ = 40.481, *P* < 0.001), all men age groups are eligible for circumcision (*X*^2^ = 45.684, *P* < 0.001), the best place to conduct SMC (*X*^2^ = 9.660, *P* = 0.002), and whether heard about SMC in the last 12 months (*X*^2^ = 6.631, *P* < 0.001), as shown in Table 3.

### 3.4. Perception of Safe Male Circumcision on HIV Risk Reduction

Most respondents (30.94%) agreed that circumcised men enjoy sex more than uncircumcised men, though the majority (28.78%) disagreed that circumcised men have more sexual feelings. A bigger percentage (28.06%) of respondents disagreed with the notion that circumcision proves manhood; however, 6.83% of the respondents strongly agreed that circumcision is an old practice that should not be reintroduced. Close to 3 in 4 (74.46%) did not know whether they would choose to be circumcised if it prevents against HIV, though a little more than three-quarters (77.34%) would accept to circumcise their boy children. Nevertheless, 10.79% of the respondents disagreed that circumcision increases penile hygiene whereas only (7.91%) accepted that circumcision reduces the risk of penile cancer as shown in Table 4.

### 3.5. Analysis of Association of Perceptions of SMC on HIV Risk Reduction and Practice of SMC

At 95% CI, individual perceptions like circumcised men have more sexual feelings (*X*^2^ = 52.527, *P* < 0.001), circumcision proves manhood (*X*^2^ = 18.032, *P* = 0.001), circumcision is an old practice and no need to reintroduce (*X*^2^ = 17.208, *P* = 0.002), whether to circumcise if it reduces HIV (*X*^2^ = 307.071, *P* < 0.001), whether to circumcise their boy children (*X*^2^ = 161.997, *P* < 0.001), and whether circumcision increases penile hygiene (*X*^2^ = 13.513, *P* < 0.001) were found significant at the bivariate analysis as shown in Table 5.

### 3.6. Practice of Safe Medical Circumcision

**Figure 1:**
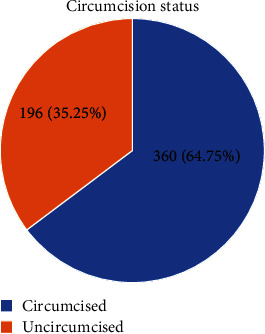
A pie chart representing the percentage number of respondents that practiced safe medical circumcision.

Majority of the respondents in this study (64.75%, 360/556) were circumcised following safe medical circumcision procedures, as shown in Figure 1.

### 3.7. Association of Sociodemographic Characteristics with Practice of Safe Medical Circumcision

A bivariate analysis between the practice of SMC and the demographic factors to determine the association at 95% CI showed that the year of study (*X*^2^ = 9.805, *P* = 0.02), religion (*X*^2^ = 35.001, *P* < 0.001), and knowing a facility where SMC is done (*X*^2^ = 29.717, *P* < 0.001) were found to be significant as shown in Table 6.

### 3.8. Sources of Information

In the analysis of the proportion of the respondents who heard about SMC in the last 12 months, health workers played a more prominent role (52.88%), yet family members were not the primary source of information for most respondents (23.38%); on the other hand, political leaders and partners had minimal influence accounting for only 7.19% and 5.04%, respectively. The source of information was found to have a reasonable influence on the respondents' decision regarding circumcision (56.12%) as shown in Table 7.

### 3.9. Bivariate Analysis on Association between the Source of Information and Practice of SMC

The analysis conducted at a 95% confidence interval showed that family influence (*X*^2^ = 11.019, *P* = 0.001) and the source of information influencing the circumcision status (*X*^2^ = 32.736, *P* < 0.001) had a statistically significant impact on the circumcision status of the respondents as shown in Table 8.

## 4. Discussion

In this study population, the majority of participants (80.58%) were within the age range of 21-25 years. This is a significant age in terms of sexual activity in males; therefore, being circumcised would provide added HIV protection to male students. Our finding conforms with results from a previous study in Nigeria where the majority of the participants were aged 20-24 years [10].

The level of SMC was 64.75% among our study participants below Uganda's target of achieving an 80% circumcision rate [11]. However, the SMC recorded in our study is higher than the 46% national circumcision rate as per the report by Uganda's Ministry of Health 2016 [12]. Also, our findings are slightly higher than the 58.3% prevalence of SMC among students of Makerere University in Central Uganda. This difference in the circumcision rates could be due to the extensive measures implemented by the Ugandan government through the Ministry of Health and related stakeholders in health to offer free safe male circumcision services to all males. Our findings are also higher than findings from a previous similar study in Eswatini, Botswana, and Rwanda where 48.98%, 47.9%, and 35.8% of the students had been circumcised, respectively [13–15]. Our finding, however, conforms with results from a study among college students in Zambia and South Africa where 63% and 78.0%, respectively, of the students were circumcised [16, 17]. In the bid to have 80% of the males circumcised in the country, concerted efforts are needed by the government through the collaboration of the Ministry of Health and Education to create awareness of the availability of free SMC services such that uptake of SMC increases among students.

In our study, majority of the study participants (81.29%) perceived HIV risk reduction when circumcised; this positive knowledge about the benefits of SMC showed a significant association with the uptake of safe male medical circumcision among university students at *X*^2^ = 307.071, *P* < 0.001. This finding is consistent with the medical reasons for providing SMC to all males. The improved awareness programs in the lower level of learning such as primary and secondary school levels could have played a significant role in providing knowledge on the benefits of circumcision among the study participants. The findings from this study agree with the results from the previous study among college students in Eswatini and Zimbabwe where all the participants perceived risk reduction in HIV contraction following medical circumcision [13, 18]. However, this HIV risk reduction perception is extremely higher than the one reported among university students in Nigeria were only 38% were aware of the role of medical circumcision in the reduction of HIV acquisition [10]. This implies the need by the Ministry of Health of Uganda to strengthen SMC programs targeting university students to optimize uptake of SMC. Involving students in awareness campaigns in learning institutions would serve as the basis for optimizing the uptake of SMC services.

In our study, 87.49% of the university students presume the health facility as the best place for conducting circumcision. Knowing/recognizing the health facility as the best place for conducting circumcision also minimizes the risk of infections including HIV transmission in the process of circumcision. This finding agrees with results from a similar study conducted in South Africa where respondents emphasized clinical setting circumcision to minimize the risk of spreading HIV while highlighting unsafe equipment in other cultural circumcision points [19]. This implies the need by the Ministry of Health of Uganda to make SMC free and readily available for university students to prevent adverse outcomes with the use of alternative ways of circumcision such as traditional surgeons, whose work may be substandard.

Furthermore, our study found that more than half (55.4%) of our participants strongly agreed that all male age groups are eligible for circumcision. This was also positively associated with SMC uptake (*X*^2^ = 45.68, *P* < 0.001). In contrast with a qualitative survey done in Northern Uganda, older participants above 30 years believed that circumcision was only for young boys and men [20]. This notion of the age of circumcision could have prompted the high circumcision rate reported in our study, given the fact that the majority (80.58%) of our participants were in the age range of 21-25 years. This implies the need for the Ministry of Health of Uganda to advocate for age-group-friendly safe medical circumcision programs such that all males in various age brackets receive safe medical circumcision services equally.

Interestingly, the majority (31.6%) of our study participants disagree with circumcised men having more sexual feelings than uncircumcised men. However, with this perception, there was a positive association with the uptake of SMC among our participants (*X*^2^ = 52.52, *P* < 0.001). The nonperceived sexual benefit difference between circumcision and noncircumcision has not limited the participants from receiving safe medical circumcision services. Our finding agrees with results from a similar study conducted in Hoima District, Western Uganda, where voluntary male medical circumcision does not diminish sexual performance (APR 1.78 (95% CI 1.08–2.9)), [21], whereas a study in Turkey assessing childhood circumcision and male sexual functioning concluded that sexual function consists of many elements that not only relate to measurable facts such as anatomy, somatosensory, and histology; thus, individuals can either perceive their circumcision status as a blessing or a curse depending on the values and preferences of the different communities or social environments where they belong [22]. This implies the desire to ally the possible misconception surrounding male circumcision if we are to achieve the target set by Uganda of achieving an 80% circumcision rate [11].

Participants highlighted the source of information about SMC to influence their circumcision status (*X*^2^ = 32.74, *P* < 0.001). Our finding is related to the article in the USA where social interactions on male circumcision influenced many respondents' decision to be circumcised though there were not formally scheduled discussions on SMC [23]. Encouraging age-appropriate health discussion plays a key role in promoting good health among people of the same age category. Therefore, for the effectiveness of certain programs such as SMC among university students, programs should be tailored based on the needs of university students such that they get fully involved in their own health care decisions.

## 5. Conclusions and Recommendation

More than one in two (64.75%) undergraduate students are circumcised. Majority of the study participants (81.29%) perceived HIV risk reduction when circumcised; this positive knowledge about the benefits of SMC showed a significant association with the uptake of safe male medical circumcision among university students. 87.49% of the university students presume the health facility as the best place for conducting circumcision. Therefore, in an effort to increase SMC's adoption for HIV/AIDS prevention, the Ministry of Health of Uganda and related stakeholders in health should work hand in hand with university study bodies in order to optimize SMC uptake among university students.

### 5.1. Study Limitations and Strength

This study was prone to social desirability bias since the data collection tool was self-administered and lacked control from the research to probe further about the participants responses. However, this limitation was mitigated by consenting and reassurance of participant privacy and confidentiality.

This study also employed quantitative methods of data collection and analysis; thus, it did not exhaust the overall feedback from the participants. Thus, a mixed method approach and both quantitative and qualitative methods of data collection and analysis are recommended for a better understanding of the phenomenon.

## Figures and Tables

**Table 1:** Sociodemographic characteristics.

Demographic characteristics	Frequency (*n*)	Percentage (%)
*Participants age*		
18-20	36	6.47
21-25	448	80.58
26-30	18	3.24
31-35	27	4.86
36-40	11	1.98
41-45	16	2.88
*Academic year of study*		
One	174	31.29
Two	184	33.09
Three	184	33.09
Four	14	2.52
*Faculty of study*		
Public health	70	12.59
Nursing and midwifery	69	12.41
Medicine	53	9.53
Computing and information science	56	10.07
Education	162	29.14
Management science	146	26.26
*Religion*		
Catholic	186	33.45
Anglican	172	30.94
Pentecostal	104	18.71
Moslem	28	5.04
None	16	2.88
Others	50	8.99
*Know facility that does SMC*		
Yes	484	87.05
No	72	12.95

**Table 2:** Knowledge of safe medical circumcision on HIV risk reduction.

Knowledge item	Frequency (*n*)	Percentage (%)
*Circumcision reduces risk of HIV*		
True	452	81.29
False	60	10.79
Not sure	44	7.91
*Circumcision of man does not protect his partner from HIV*		
True	312	56.12
False	162	29.14
Not sure	82	14.75
*It is easy to get HIV when uncircumcised*		
No	16	2.88
Yes	476	85.61
Do not know	64	11.51
*Circumcised men can have unprotected sex and do not get HIV*		
Strongly agree	80	14.39
Agree	162	29.14
No option	50	8.99
Disagree	174	31.29
Strongly disagree	90	16.19
*All men age groups are eligible for circumcision*		
Strongly agree	308	55.4
Agree	154	27.7
No option	48	8.63
Disagree	28	5.04
Strongly disagree	18	3.24
*Best place to conduct circumcision*		
Home	40	7.19
Health facility	492	88.49
No preference	12	2.16
Do not know	12	2.16
*Heard of circumcision in the past 12* months		
Yes	472	84.89
No	84	15.11
*Reasons for circumcision*		
Religion	38	6.83
Cultural norms	38	6.83
Hygiene	224	40.29
Others	60	10.79
Not required	196	35.25

**Table 3:** Association of SMC knowledge on HIV risk reduction and practice of SMC.

Knowledge item	Practiced SMC		Chi-square	*P* value
	Yes, *n* (%)	No, *n* (%)		
*Circumcision reduces risk of HIV*				
True	308 (85.6)	144 (73.5)	18.404	<0.001^∗^
False	36 (10.0)	24 (12.2)		
Not sure	16 (4.4)	28 (14.3)		
*Circumcision of man does not protect his partner from HIV*				
True	186 (51.7)	126 (64.3)	17.037	<0.001^∗^
False	126 (35.0)	36 (18.4)		
Not sure	48 (13.3)	34 (17.4)		
*It is easy to get HIV when uncircumcised*				
No	10 (2.8)	6 (3.1)	18.650	<0.001^∗^
Yes	324 (90.0)	152 (77.6)		
Do not know	26 (7.2)	38 (19.4)		
*Circumcised men can have live sex and do not get HIV*				
Strongly agree	122 (33.9)	36 (18.4)	40.481	<0.001^∗^
Agree	126 (35.0)	46 (23.5)		
No option	44 (12.2)	42 (21.4)		
Disagree	42 (11.7)	38 (19.4)		
Strongly disagree	26 (7.2)	34 (17.4)		
*All men age groups are eligible for circumcision*				
Strongly agree	228 (63.3)	80 (40.8)	45.684	<0.001^∗^
Agree	94 (26.1)	60 (30.6)		
No option	24 (6.7)	24 (12.3)		
Disagree	12 (3.3)	16 (8.2)		
Strongly disagree	2 (0.6)	16 (8.2)		
*Best place to conduct circumcision*				
Home	34 (9.4)	6 (3.1)	9.660	0.021^∗^
Health facility	314 (87.2)	178 (90.8)		
No preference	6 (1.7)	6 (3.1)		
Do not know	6 (1.7)	6 (3.1)		
*Heard of circumcision in the past 12 months*				
Yes	316 (87.9)	156 (79.6)	6.631	0.010^∗^
No	44 (12.2)	40 (20.4)		

^∗^Significant at 95% confidence interval.

**Table 4:** Perception about safe medical circumcision on HIV risk reduction.

Perception item	Frequency (*n*)	Percentage (%)
*Circumcised men enjoy sex than uncircumcised*		
Strongly agree	158	28.42
Agree	172	30.94
No option	86	15.47
Disagree	80	14.39
Strongly disagree	60	10.79
*Circumcised men have more sexual feelings*		
Strongly agree	86	15.47
Agree	128	23.02
No option	124	22.3
Disagree	160	28.78
Strongly disagree	58	10.43
*Circumcision proves manhood*		
Strongly agree	52	9.35
Agree	82	14.75
No option	112	20.14
Disagree	156	28.06
Strongly disagree	154	27.7
*Circumcision is an old practice and no need to reintroduce*		
Strongly agree	38	6.83
Agree	38	6.83
No option	96	17.27
Disagree	176	31.65
Strongly disagree	208	37.41
*Would you circumcise if it prevents infection*		
Yes	74	13.31
No	68	12.23
Do not know	414	74.46
*Would you circumcise your boy child*		
Yes	430	77.34
No	58	10.43
Do not know	68	12.23
*Circumcision increases penile hygiene*		
Yes	496	89.21
No	60	10.79
*Circumcision reduces risk of penile cancer*		
Yes	44	7.91
No	512	92.09

**Table 5:** Association of perception of SMC on HIV risk reduction and practice of SMC.

Perception item	Practiced of SMC		Chi-square	*P* value
	Yes, *n* (%)	No, *n* (%)		
*Circumcised men have more sexual feelings*				
Strongly agree	72 (20.0)	14 (7.1)	52.527	<0.001^∗^
Agree	98 (27.2)	30 (15.3)		
No option	74 (20.6)	50 (25.5)		
Disagree	98 (27.8)	62 (31.6)		
Strongly disagree	18 (5.0)	40 (20.4)		
*Circumcision proves manhood*				
Strongly agree	36 (10.0)	16 (8.2)	18.032	0.001^∗^
Agree	62 (17.2)	20 (10.2)		
No option	72 (20.0)	40 (20.4)		
Disagree	110 (30.6)	46 (23.5)		
Strongly disagree	80 (22.2)	74 (37.8)		
*Circumcision is an old practice and no need to reintroduce*				
Strongly agree	16 (4.4)	22 (11.2)	17.280	0.002^∗^
Agree	28 (7.8)	10 (5.1)		
No option	16 (17.2)	34 (17.4)		
Disagree	104 (28.9)	72 (36.7)		
Strongly disagree	150 (41.7)	58 (29.6)		
*Would you circumcise if it reduces the risk of infection*				
Yes	6 (1.7)	68 (34.7)	307.071	<0.001^∗^
No	0 (0)	68 (34.7)		
Do not know	354 (98.3)	60 (30.6)		
*Would you circumcise your boy child?*				
Yes	338 (93.9)	92 (46.9)	161.997	<0.001^∗^
No	6 (1.7)	52 (26.5)		
Do not know	16 (4.4)	52 (26.5)		
*Circumcision increases penile hygiene*				
Yes	334 (92.8)	162 (82.7)	13.513	<0.001^∗^
No	26 (7.2)	34 (17.4)		
*Circumcision reduces the risk of penile cancer*				
Yes	26 (7.2)	18 (9.2)	0.670	0.411
No	334 (92.8)	178 (90.8)		

^∗^Significant at 95% confidence interval.

**Table 6:** Association of sociodemographic characteristics with practice of safe medical circumcision.

Factor	SMC practice		Chi-square	*P* value
	Yes, *n* (%)	No, *n* (%)		
*Participants' age*				
18-20	18 (5.0)	18 (9.2)	4.613	0.470
21-25	292 (81.1)	156 (79.6)		
26-30	12 (3.3)	6 (3.1)		
31-35	19 (5.3)	8 (4.1)		
36-40	7 (1.9)	4 (2.0)		
41-45	12 (3.3)	4 (2.0)		
*Academic year of study*				
One	100 (27.8)	74 (37.8)	9.805	0.020^∗^
Two	116 (32.2)	68 (34.7)		
Three	134 (37.2)	50 (25.5)		
Four	10 (2.8)	4 (2.0)		
*Faculty*				
Public health	40 (11.1)	30 (15.3)	16.604	0.050^∗^
Nursing and midwifery	50 (13.9)	19 (9.7)		
Medicine	41 (11.4)	12 (6.1)		
Computing and information science	33 (9.2)	23 (11.7)		
Education	91 (25.3)	71 (36.2)		
Management science	105 (29.2)	41 (20.9)		
*Religion*				
Catholic	142 (39.4)	34 (22.5)	35.001	<0.001^∗^
Anglican	109 (30.0)	64 (32.7)		
Pentecostal	50 (13.9)	54 (27.6)		
Moslem	24 (6.7)	4 (2.0)		
None	6 (1.7)	10 (5.1)		
Others	30 (8.3)	20 (10.2)		
*Know facility that does SMC*				
Yes	334 (92.8)	150 (76.5)	29.717	<0.001^∗^
No	26 (7.2)	46 (23.5)		

^∗^Significant at 95% confidence interval.

**Table 7:** Source of information.

Sources of information about SMC	Frequency (*n*)	Percentage (%)
*Family*		
Yes	130	23.38
No	426	76.62
*Partner*		
Yes	28	5.04
No	528	94.96
*Friend*		
Yes	196	35.25
No	360	64.75
*Health worker*		
Yes	294	52.88
No	262	47.12
*Political leader*		
Yes	40	7.19
No	516	92.81
*Traditional leader*		
Yes	30	5.4
No	526	94.6
*Other sources*		
Yes	232	41.73
No	324	58.27
*Did the source influence circumcision*		
Yes	312	56.12
No	244	43.88

**Table 8:** Association of source of information with SMC practice.

Sources of information about SMC	SMC practice		Chi-square	*P* value
	Yes, *n* (%)	No, *n* (%)		
*Family*				
Yes	100 (27.8)	30 (15.3)	11.019	0.001^∗^
No	260 (72.2)	166 (84.7)		
*Partner*				
Yes	20 (5.6)	8 (4.1)	0.577	0.450
No	340 (94.4)	188 (96.0)		
*Friend*				
Yes	132 (36.7)	64 (32.7)	0.896	0.340
No	228 (63.3)	132 (67.4)		
*Health worker*				
Yes	200 (55.6)	94 (48.0)	2.940	0.091
No	160 (44.4)	102 (52.0)		
*Political leader*				
Yes	34 (9.4)	6 (3.1)	7.741	0.005^∗^
No	326 (90.6)	190 (96.9)		
*Traditional leader*				
Yes	22 (6.1)	8 (4.1)	1.024	0.311
No	338 (93.9)	188 (95.9)		
*Other sources*				
Yes	20 (5.6)	14 (7.1)	0.557	0.463
No	340 (94.4)	182 (92.9)		
*Did the source influence circumcision*				
Yes	234 (65.0)	78 (39.8)	32.736	<0.001^∗^
No	126 (35.0)	118 (60.2)		

^∗^Significant at 95% confidence interval.

## Data Availability

The data sets used and/or analyzed during the study are available from the corresponding author on reasonable request.
